# Assessment of machine perfusion conditions for the donation after circulatory death heart preservation

**DOI:** 10.1111/aor.14208

**Published:** 2022-02-22

**Authors:** Renee Cholyway, Oluwatoyin Akande, Adolfo Gabriele Mauro, Eleonora Mezzaroma, Rui Wang, Kristine Kenning, Stefano Toldo, Mohammed Quader

**Affiliations:** ^1^ Division of Thoracic and Cardiovascular Surgery, Department of Surgery Virginia Commonwealth University Richmond Virginia USA; ^2^ Pauley Heart Center Virginia Commonwealth University Health System Richmond Virginia USA; ^3^ Division of Cardiology, Department of Internal Medicine Virginia Commonwealth University Richmond Virginia USA; ^4^ Department of Pharmacotherapy and Outcome Science, School of Pharmacy Virginia Commonwealth University Richmond Virginia USA; ^5^ McGuire Veterans Administration Medical Center Richmond Virginia USA

**Keywords:** cardiac transplantation, donation after circulatory death heart, heart preservation, machine perfusion preservation, myocardial perfusion

## Abstract

**Background:**

Donation after circulatory death (DCD) hearts requires machine perfusion preservation, the conditions of which are not well defined.

**Methods:**

To achieve this, rat hearts were procured following a DCD or control beating‐heart donation (CBD) model, and perfused for 60 min with one of three machine perfusion solutions—St. Thomas (ST), University of Wisconsin (UW), or Polyethylene Glycol‐20k (PEG)—at one of two temperatures, 4°C or 15°C. At 15‐min intervals, perfusion pressure was measured as a marker of vascular resistance. Colored microspheres were added to capture the distribution of perfusate into the metabolically active sub‐endocardium, and the eluate was collected for troponin assays. Analyses compared groups using Wilcoxon rank‐sum and ANOVA.

**Results:**

Perfusion pressure was significantly higher for DCD than CBD hearts at 15°C regardless of solutions. The lowest rise in perfusion pressure over time was observed with PEG at 15°C. Except for PEG at 15°C, ST and UW solutions at 4 or 15°C had decreased sub‐endocardial perfusion in DCD hearts. Troponin release from DCD hearts with UW and PEG solutions was comparable to CBD hearts but was significantly higher with ST solution at 15°C.

**Conclusions:**

Optimal preservation conditions for DCD hearts were observed with PEG machine perfusion solution at 15°C.

## Graphical abstract

Donation after circulatory death (DCD) hearts requires machine perfusion preservation, but the conditions are not empirically defined. DCD rat hearts, or control hearts, were procured and perfused for 60 min with one of three machine perfusions solutions (MPS)—St. Thomas (ST), University of Wisconsin (UW), or Polyethylene Glycol‐20k (PEG)—at one of two temperatures—4°C or 15°C. Compared to the other solutions and temperature, perfusion preservation with PEG MPS at 15°C, displayed a homogeneous tissue perfusion and reduced cardiac damage.

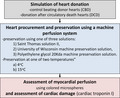

## 1 BACKGROUND

Approximately 10% of patients with advanced heart failure will require heart transplantation in the United States.^1^, ^2^, ^3^ Currently, the donor hearts primarily come from patients with complete and irreversible brain damage (Donation after Brain Death, DBD), but a disproportionate increase in potential recipients outnumbers the supply due to improved medical and device management.^2^, ^4^, ^5^, ^6^ Thus, there is an urgent need to expand the heart donor pool. A potential source of donor hearts is Donation after Circulatory Death (DCD) donors, these are patients with a terminal illness or with severe irreversible brain damage who has some residual brain stem activity that precludes designation “brain dead”.^4^, ^5^, ^7^ Utilizing DCD hearts could increase the number of heart transplants by up to 56%, significantly decrease the mortality of patients on the waitlist, and shorten the wait times for all patients.^8^, ^9^, ^10^, ^11^

Unlike the DBD hearts, the DCD hearts sustain significant warm ischemic damage and do not maintain viability with cold storage for transportation.^12^ Reperfusion injury is also a well‐established phenomenon, initiated at the moment of transplantation or ex situ organ perfusion.^13^, ^14^ Current practice for DCD transplantation uses whole blood for organ preservation ex situ. However, using blood as perfusate has limitations such as securing adequate blood for perfusion and hemolysis from roller pumps that would plug the capillaries. In addition, the donor blood carries cytokines and high levels of catecholamines that can damage the DCD heart.^15^

Oxygenated non‐blood‐based organ preservation solutions can mitigate these shortcomings by blunting the deleterious effects of reperfusion and tissue edema while providing protection from ongoing ischemia and allowing for assessment of cardiac function before transplantation. The most commonly used organ preservation solutions are the University of Wisconsin machine perfusion solution (UW‐MPS) and the Saint Thomas solution‐2 (ST). While UW and ST solutions have an excellent record in preserving DBD hearts, they were not well studied in DCD heart preservation. A modified UW‐MPS with added 20KDa polyethylene glycol (PEG), a better cell impermeant that limits tissue edema and improves intravascular volume, has been shown to be a superior organ preservative solution. Constituents and comparison of three solution used in our study are shown in Table 1.^16^

**TABLE 1:** Composition of perfusate solutions

Perfusate solutions composition	ST. Thomas (ST)	University of Wisconsin (UW)	Polyethylene glycol‐20k (PEG)
Chemical name	Concentration		
HES (Starch)		5% (w/v)	
PEG‐20kDa			2.5% (w/v)
Glucose	11 mM	10 mM	10 mM
Na‐Octanoate			1 mM
NaCl	110 mM		
NaHCO_3_	10 mM		
KCl	16 mM		
MgCl_2_	16 mM		
CaCl_2_	1.2 mM	0.5 mM	0.5 mM
KH_2_PO_4_		15 mM	15 mM
K‐Gluconate		10 mM	
Mg‐Gluconate		5 mM	5 mM
Ribose		5 mM	
Adenine		5 mM	
Adenosine			5 mM
HEPES		10 mM	10 mM
Allopurinol		1 mM	1 mM
Glutathione		3 mM	3 mM

Amino acids	Concentration (g/L)		
l‐Arginine (free base)			10.0
l‐Asparagine•H_2_O			2.84
l‐Aspartic acid			1.0
l‐Cystine			2.5
l‐Glutamic acid			1.0
l‐Glutamine			0.29
Glycine			0.5
l‐Histidine (free base)			0.75
Hydroxy‐l‐proline			1.0
l‐Isoleucine			2.5
l‐Leucine			2.5
l‐Lysine•HCl			2.0
l‐Methionine			0.75
l‐Phenylalanine			0.75
l‐Proline			1.0
l‐Serine			1.5
l‐Threonine			1.0
l‐Tryptophan			0.25
l‐Tyrosine			1.16
l‐Valine			1.0
pH	7.8	7.4	7.4

Unlike the DBD hearts, DCD hearts preserved on a machine perfusion system required a higher perfusion rate and may require a lower temperature to maintain the heart in an aerobic state with non‐blood‐based perfusate.^17^ Extreme hypothermic conditions (<4°C) are known to inflict endothelial damage and produce a lower ATP generation per oxygen consumed upon reperfusion as compared to midthermic temperature (21°C).^6^, ^18^, ^19^ Studies support mid‐thermic temperature (roughly 12–21°C) for organ preservation to provide the best protection against reperfusion injury.^18^, ^19^, ^20^, ^21^

The goal of this study is to identify an ideal non‐blood‐based machine perfusion condition including temperature and solution for a DCD heart that ensures homogeneous myocardial perfusion over time and superior myocardial preservation. We hypothesize that there is a difference between the extent of homogenous perfusion of metabolically active and ischemia‐sensitive sub‐endocardium among the three preservation solutions and that the heart is better preserved at a mid‐thermic temperature (15°C) compared to hypothermic temperature (4°C).

## 2 METHODS

These experiments were conducted under the guidelines of the “Guide for the care and use of laboratory animals” published by the National Institutes of Health.^22^ The study protocol was approved by the Virginia Commonwealth Institutional Animal Care and Use Committee.

### 2.1 Groups

**FIGURE 1:**
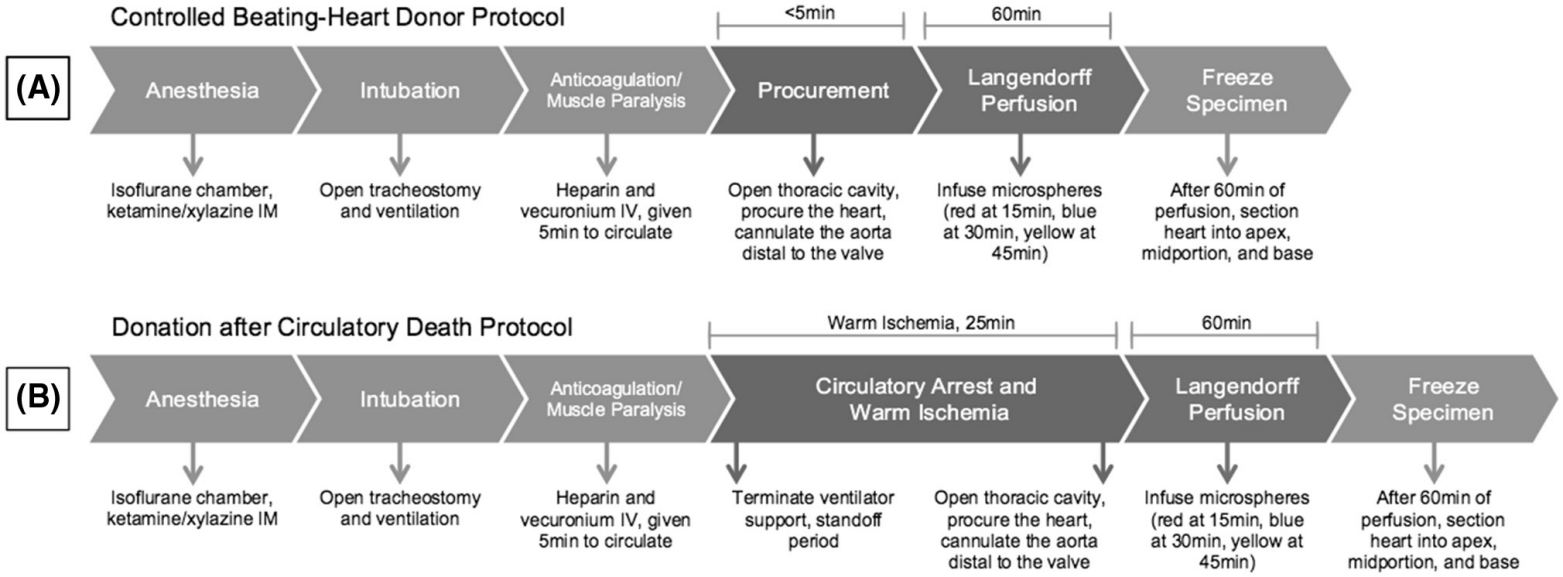
Schematic representation of the (A) controlled beating‐heart donor (CBD) and (B) donor after circulatory death (DCD) protocols

Seventy‐two Sprague–Dawley rats of 10–12 weeks old were assigned half to controlled beating‐heart donor (CBD) and the other half to a DCD protocol as published before (Figure 1).^23^ The 36 hearts in each protocol were assigned for perfusion with one of the three machine perfusion solutions (ST, UW, or PEG) at two temperatures (4°C or 15°C) to create six groups: six hearts were perfused with Saint Thomas (ST) solution at 15°C, six with ST at 4°C, six with University of Wisconsin (UW) solution at 15°C, six with UW at 4°C, six with polyethylene glycol‐20k (PEG) solution at 15°C, and the six with PEG at 4°C (Table 2).

**TABLE 2:** Conditions for perfusion and number of rat hearts

Condition	Perfusate	Temperature	
		15°C	4°C
CBD (control)	ST	6	6
CBD (control)	UW	6	6
CBD (control)	PEG	6	6
DCD	ST	6	6
DCD	UW	6	6
DCD	PEG	6	6
Total number of hearts		36	36

Abbreviations: CBD, controlled beating‐heart donor; DCD, donor after circulatory death; PEG, polyethylene glycol‐20k machine perfusion solution; ST, St. Thomas; UW, University of Wisconsin machine perfusion solution.

Rats were anesthetized by administering ketamine/xylazine (100/10 mg/kg) intramuscularly, followed by endotracheal intubation for respiratory support. The depth of anesthesia was monitored by checking for the absence of pedal reflexes. Heparin (400 Units) and vecuronium bromide (2 mg) were administered through the right internal jugular vein and allowed to circulate for 5 min. For the DCD model, the animals were then released from ventilator support to induce respiratory arrest leading to cardiac asystole.^23^ Thoracotomy was performed and hearts procured to be perfused via cannula attached to the ascending aorta on Langendorff setup. The total time from ventilation withdrawal to initiating reperfusion on the Langendorff apparatus was standardized to 25 min. The control CBD rats were ventilated until the moment of heart procurement; hearts were then mounted on the Langendorff system similar to the DCD hearts. Time from procurement to perfusion of the control heart was less than 5 min.

### 2.2 Perfusion

**FIGURE 2:**
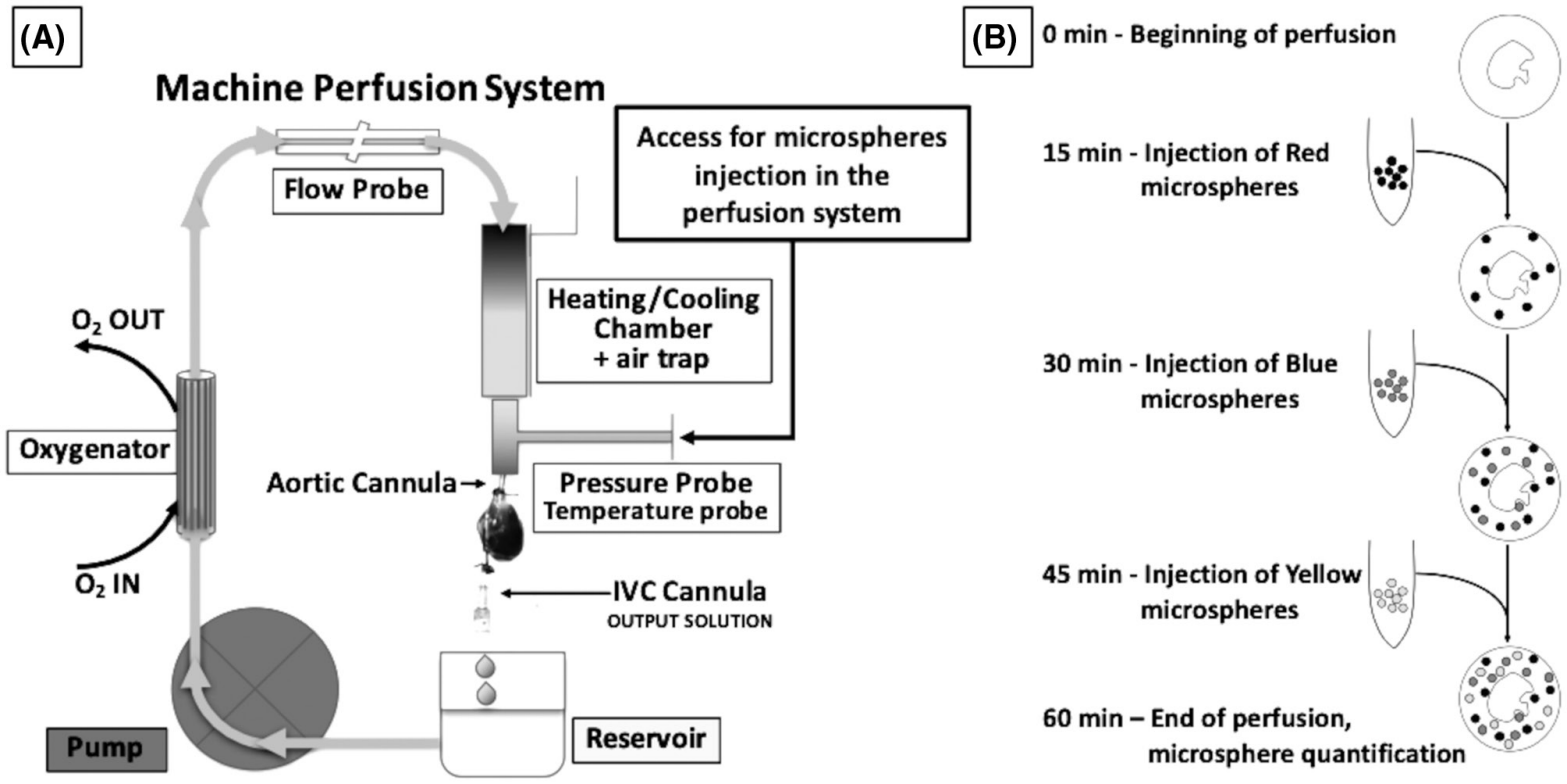
Schemes of the machine perfusion system and of the microspheres distribution study. Major components of the machine perfusion system (panel A) are the peristaltic pump, the oxygenator, the flow probe, the chamber for temperature regulation, a pressure probe, a temperature probe and a three‐way stopcock to add the colored microspheres into the column of perfusion solution, above the heart. The heart is antegradely perfused through the aorta. The solution drains through the inferior vena cava (IVC) for the measurement of chemical/physical parameters. In the colored microspheres study (panel B), microspheres of different color will be injected into the perfusion system at constant intervals. Red microspheres will be injected into the system after 15 min of perfusion and will be allowed to distribute and house in the microvasculature. At 30 min, when all the red microspheres are trapped in the tissue, the blue microspheres will be injected, followed, after 45 min, by the yellow microspheres. After a total of 60 min, the hearts are collected to measure the microsphere distribution in left ventricular slides

The Langendorff perfusion system was primed with 200 ml of one of the three organ preservation solutions (ST, UW, and PEG), and set to run in a closed circuit at a fixed flow of 140–170 ml/100 g/min for 60 min at one of the two temperatures, 4°C or 15°C. The three solutions used are cardioplegic and therefore prevent the heart beat and lowers the ATP consumption during organ preservation. The perfusion flow at the selected temperatures was chosen based on prior studies performed to determine the critical flow needed to maintain a DCD heart in aerobic condition during preservation.^17^, ^24^ Heart weight was measured to adjust for perfusion flow. Mean weight of heart and standard deviation were 1.34 ± 0.16 and 1.32 ± 0.21 g in the CBD and DCD groups, respectively. A schematic representation of the perfusion system used, is reported in Figure 2A. Colored DYE‐TRAK® microspheres were used to track the regional tissue flow. These microspheres have a diameter of 12 microns and are designed to distribute with the flow of blood (or other perfusates), and get dispersed and trapped in the terminal microvasculature, which has an average diameter of 5 microns, too small for the microspheres to pass through. Approximately 50 000 colored microspheres (12‐micron diameter) were added to 3 ml of respective perfusate, agitated, and delivered via three‐way stopcock into the perfusion system, directly into the column above the aortic cannula providing perfusion. (Figure 2A). To determine whether the perfusion pattern changes over the 60 min of perfusion, we delivered different colored microspheres at 15 min interval. From the onset of perfusion to 15 min red color microspheres (Persimmon DYE‐TRAK® VII+, Cat# 145‐0545, Triton Technology Inc., San Diego, CA, USA) were injected, at 30 min, blue color microspheres (Blue DYE‐TRAK® microspheres, Cat# 145‐0672, Triton Technology Inc.) were injected, and at 45 min the yellow color microspheres (Yellow DYE‐TRAK® VII+ microspheres, Cat# 145‐0448, Triton Technology Inc.) were injected and allowed to distribute for 15 min. Figure 2B shows a diagram of the timing and colored microspheres administration, which, thanks to the use of different colors, reflects the flow distribution at the precise moment the microspheres were trapped while moving through the vasculature. Concurrently at 15, 30, 45, and 60 min we gathered information on the temperature, flow rate, and perfusion pressures with catheters connected to a Powerlab‐station and Labchart‐7 (AD Instruments, Colorado Springs CO). Eluate drained from the coronary sinus was collected for cardiac troponin I release as a marker of myocyte injury, quantified using the rat cardiac Troponin I (cTnI) ELISA Kit (Cat No. R6691, TSZ Scientific). At the end of 60 min of perfusion, the hearts were sliced along the short axis into an apex, mid‐portion, and base then frozen. Three slides from each section were cut with a cryostat at 10 μm thickness for a total of nine slides per heart: three from the apex, three from the mid‐portion, and three from the base. Each slide was stained with eosin and photographed under 10× magnification, with a line drawn to delineate the left ventricular myocardium (~80% volume of the left ventricle) from the metabolically active ischemia‐sensitive sub‐endocardium (~20% volume of the left ventricle). The color and quantity of microspheres within the myocardium and the sub‐endocardium were counted and recorded.

### 2.3 Statistics

Median (IQR 25%, 75%) or mean (SD) is compared based on the distribution of data. Non‐parametric Wilcoxon rank‐sum test is used for comparisons, then Steel‐Dwass each pair for comparisons including more than two groups, reporting the *p*‐value. For data with normal distribution, equal variance ANOVA followed by Tukey HSD is performed for comparisons between more than two groups with *p*‐value reported. Data outliers due to technical errors (8 CBD hearts from different groups, and 5 DCD hearts from different groups) were excluded from the microsphere count analysis to prevent artificially skewed data. Significance was set to a *p*‐value of <0.05. JMP (SAS Institute Inc. Version 15) statistical software was used for calculations. All raw data generated from this study are available upon request.

## 3 RESULTS

### 3.1 Coronary vascular resistance

**FIGURE 3:**
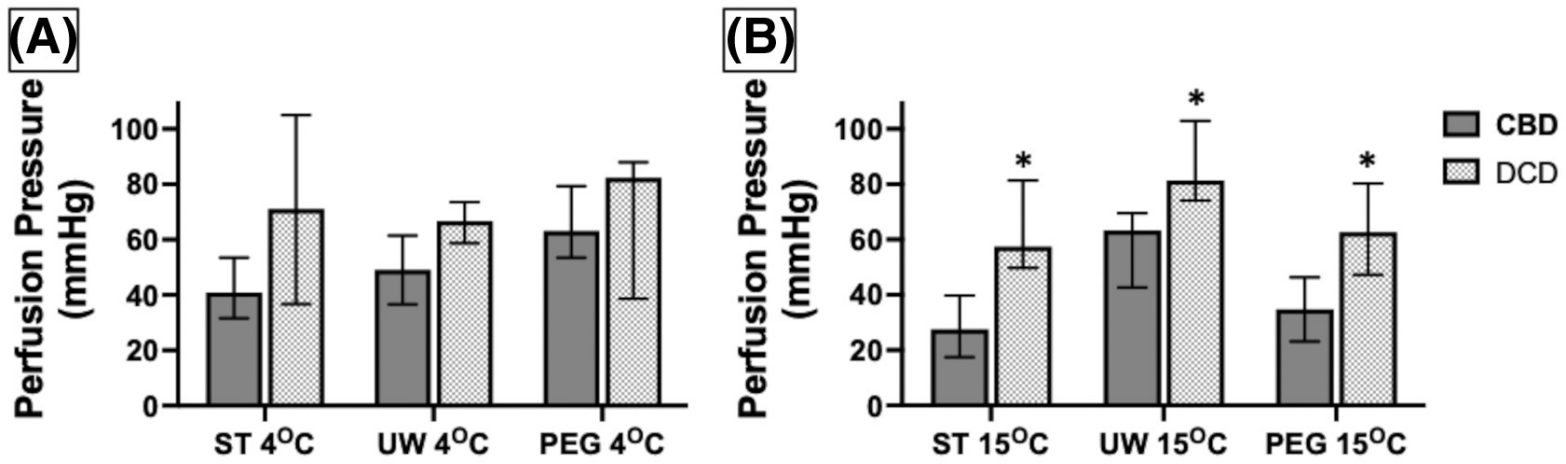
Coronary vascular resistance, demonstrated by perfusion pressure, (A) displayed at 4°C and (B) at 15°C. CBD, controlled beating‐heart donor; DCD, donation after circulatory death; PEG, polyethylene glycol 20k solution; ST, ST. Thomas solution; UW, University of Wisconsin solution. Data are expressed as median perfusion pressure. Number of samples per group: CBD ST 4°C *n* = 6, UW 4°C *n* = 6, PEG 4°C *n* = 6, ST 15°C *n* = 6, UW 15°C *n* = 6, PEG 15°C *n* = 6. DCD ST 4°C *n* = 6, UW 4°C *n* = 6, PEG 4°C *n* = 6, ST 15°C *n* = 6, UW 15°C *n* = 6, PEG 15°C *n* = 6. The statistical method used was non‐parametric Wilcoxon rank‐sum test followed by Steel‐Dwass each pair when three groups were compared, and *p* < 0.05 considered significant denoted with an (*). CBD vs. DCD 4°C ST (*p* = 0.297), UW (*p* = 0.055), PEG (*p* = 0.337). CBD vs. DCD at 15°C using ST (*p* = 0.007), UW (*p* = 0.01), PEG (*p* = 0.01). DCD ST vs. UW vs. PEG at 4°C (*p* = 0.849) and 15°C (*p* = 0.119). DCD 4°C vs. 15°C ST (*p* = 0.749), UW (*p* = 0.016), and PEG (*p* = 0.575)

Perfusion pressure at the fixed target flow rate was used as a surrogate for coronary vascular resistance. The average perfusion pressure was calculated for each group from measurements obtained at 15, 30, 45, and 60 min of perfusion. The overall median values were compared between CBD and DCD hearts perfused with the same solution (ST, UW, or PEG) at 4 and 15°C. In addition, within the DCD group, the coronary vascular resistance was studied with the three solutions at 4 and 15°C, and with the same solution comparing perfusion pressure at 4 and 15°C (Figure 3). For temperature and perfusate conditions, all DCD hearts had elevated perfusion pressures compared to CBD hearts; however, the difference was statistically significant only at 15°C (ST *p* = 0.007, UW *p* = 0.01, and PEG *p* = 0.01). Within the DCD groups, there was no significant difference in perfusion pressure with ST, PEG, or UW solutions at either 4°C (*p* = 0.278) or at 15°C (*p* = 0.160). Except for the UW solution, DCD hearts had no significant change in perfusion pressure with ST or PEG between 4 and 15°C. UW solution had higher perfusion pressure at 15°C compared to 4°C (*p* = 0.016). These results suggest that the ischemic damage to the DCD hearts likely causes microvascular injury leading to tissue edema and high vascular resistance, especially at 15°C. Within the DCD groups, there was no significant difference in perfusion pressure with any of the three solutions.

**FIGURE 4:**
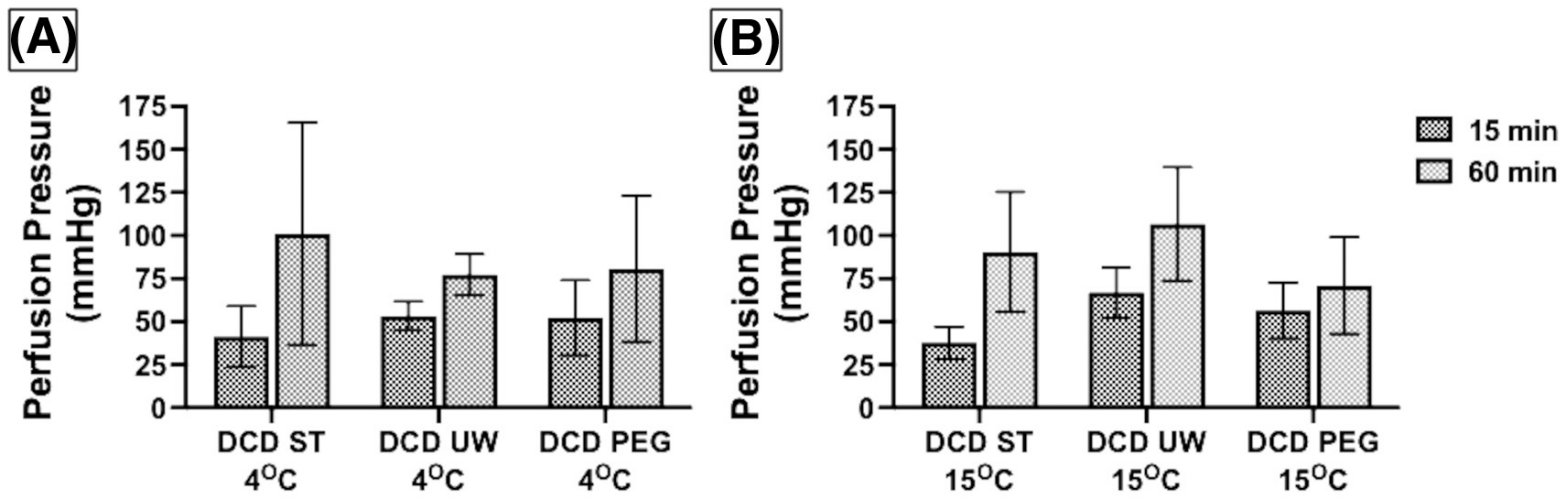
Rise in coronary vascular resistance, demonstrated by the change in perfusion pressure from 15 min perfusion to 60 min perfusion, increased with continued perfusion regardless of solution or temperature but no comparisons were statistically significant. This trend is displayed in (A) at 4°C and (B) at 15°C. DCD, donation after circulatory death; PEG, polyethylene glycol 20k solution; ST, ST. Thomas solution; UW, University of Wisconsin Belzer solution. Data is expressed as mean perfusion pressure measured after 15 min of perfusion and after 60 min of perfusion. Number of samples per group: DCD ST 4°C *n* = 6, UW 4°C *n* = 6, PEG 4°C *n* = 6, ST 15°C *n* = 6, UW 15°C *n* = 6, PEG 15°C *n* = 6. The statistical method used was non‐parametric Wilcoxon rank‐sum test (4° comparison: *p* = 0.278, and 15° comparison: *p* = 0.16), and *p* < 0.05 considered significant

To study the change in perfusion pressure with time, we collected data on the perfusion pressures measured at the end of 15 min and compared them to the pressure measured at 60 min of perfusion. We collected this data with the three solutions at 4 and 15°C. The lowest median rise in perfusion pressure was seen for PEG at 15°C and highest median rise in pressures was seen with ST solution. However, these pressure increases were not statistically significant (Figure 4). An increase in perfusion pressure over time likely suggests ongoing tissue ischemia–reperfusion injury, swelling, and increased vascular resistance. A solution with the least rise in perfusion pressure over time might be a better preservative for the heart. Our data showed a better cardioprotection trend with PEG solution at 15°C, but the difference did not reach statistical significance.

### 3.2 Microsphere distribution

**FIGURE 5:**
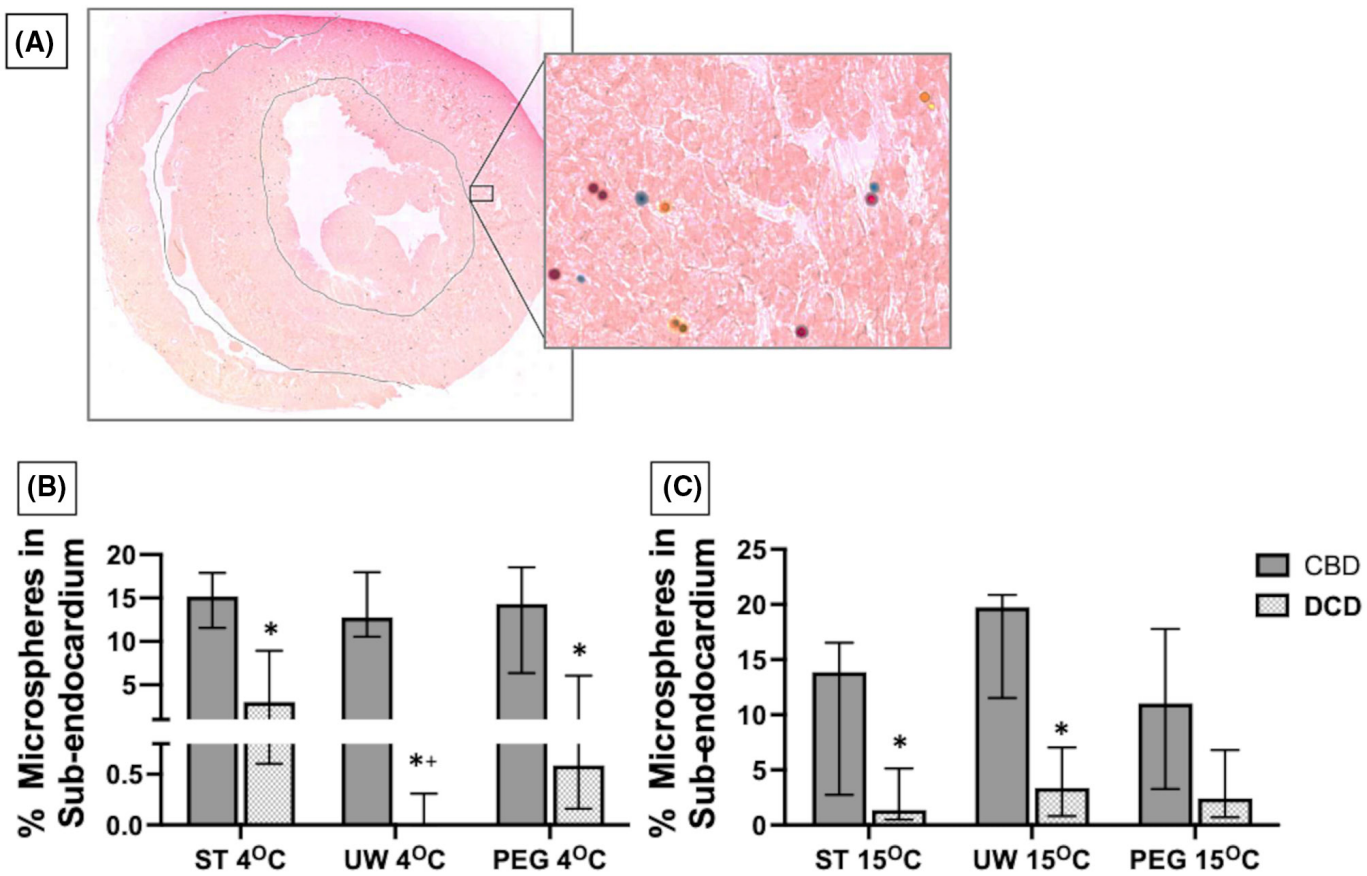
Colored microspheres distribution in the myocardium under 10‐× magnification (panel A). The portion of microspheres perfused into the sub‐endocardial space vs. myocardial space significantly decreased in all DCD hearts compared to CBD hearts, except when using PEG solution at 15°C. this is displayed visually as the percent of the total amount of counted microspheres of all colors in the sub‐endocardial space with comparisons at 4°C (panel B) and at 15°C (panel C). CBD, controlled beating‐heart donor; DCD, donation after circulatory death; PEG, polyethylene glycol 20k solution; ST, ST. Thomas solution; UW, University of Wisconsin Belzer solution. Data compared the median ratio of all colored microspheres in the sub‐endocardial to myocardial space. Number of samples per group: CBD ST 4°C *n* = 4, UW 4°C *n* = 5, PEG 4°C *n* = 6, ST 15°C *n* = 4, UW 15°C *n* = 5, PEG 15°C *n* = 4. DCD ST 4°C *n* = 5, UW 4°C *n* = 6, PEG 4°C *n* = 5, ST 15°C *n* = 6, UW 15°C *n* = 6, PEG 15°C *n* = 3. The statistical method used was non‐parametric Wilcoxon rank‐sum test followed by Wilcoxon each pair when three groups were compared, and *p* < 0.05 considered significant denoted with an (*) for CBD vs. DCD comparison and (†) for DCD solution comparison. CBD vs. DCD 4°C ST (*p* = 0.011), UW (*p* = 0.003), PEG (*p* = 0.029). CBD vs. DCD at 15°C using ST (*p* = 0.037), UW (*p* = 0.01), PEG (*p* = 0.202). DCD ST vs. UW vs. PEG at 4°C (*p* = 0.009; UW vs. ST *p* = 0.004, UW vs. PEG *p* = 0.044, ST vs. PEG *p* = 0.411) and 15°C (*p* = 0.83). DCD 4°C vs. 15°C ST (*p* = 0.522), UW (*p* = 0.005), and PEG (*p* = 0.273)

The ratio of colored microspheres perfused deeply into the sub‐endocardial space to the myocardial space (E/M ratio) was used as a visible surrogate for even distribution of perfusate. The E/M ratio is displayed as a percent of microspheres perfused into the sub‐endocardium from the total amount of microspheres counted (Figure 5A). The overall median E/M ratios were compared between CBD and DCD hearts using the same solution at 4 and 15°C, between DCD hearts using the three solutions at 4 and 15°C, and with the same solution comparing 4 and 15°C (Figure 5B,C). Compared to CBD hearts, all DCD hearts perfused a significantly lower proportion of microspheres into the sub‐endocardial tissue except for those perfused with PEG solution at 15°C (*p* = 0.2). In comparing the three solutions for DCD hearts at 4°C, DCD hearts perfused with UW had a significantly lower portion of microspheres into the sub‐endocardial space compared with PEG and ST (*p* = 0.009) because the median E/M ratio from hearts with UW was 0 (IQR 0, 0.002). There was no difference in the E/M ratio between the three solutions when examined at 15°C (*p* = 0.83). The E/M ratio compared at 4°C versus 15°C for the three solutions was comparable except when using UW solution (*p* = 0.005), likely due to a particularly low median and IQR for UW hearts at 4°C.

**FIGURE 6:**
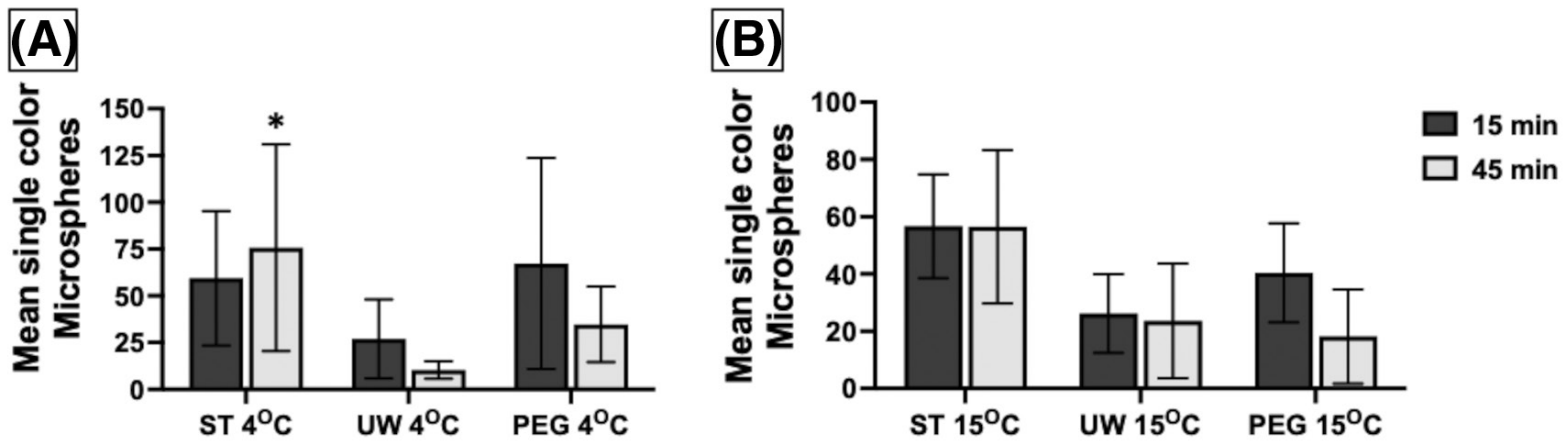
Mean total amount of single color microspheres increased with continued perfusion in DCD hearts at 4° with ST differing significantly from the decrease seen in DCD hearts at 4° with UW (*p* = 0.037), seen in (A). No difference at 15°C seen in (B). DCD, donation after circulatory death; PEG, polyethylene glycol 20k solution; ST, ST. Thomas solution; UW, University of Wisconsin Belzer solution. Data is expressed as mean total amount of microspheres after 15 min of perfusion (red microspheres) and after 45 min of perfusion (blue microspheres). Number of samples per group: DCD 4°PEG *n* = 6, 4°ST *n* = 5, 4°UW *n* = 6, 15°ST *n* = 6, 15°PEG *n* = 5, 15°UW *n* = 6. The statistical method used was equal variance ANOVA followed by Tukey HSD each pair comparison, and *p* < 0.05 considered significant designed with (*). DCD ST vs. UW vs. PEG at 4°C (*p* = 0.037); ST vs. PEG (*p* = 0.134), UW vs. PEG (*p* = 0.832), ST vs. UW (*p* = 0.0371). DCD ST vs. UW vs. PEG at 15°C (*p* = 0.273)

Along with an increased coronary vascular resistance, a reduction in perfusion rate was anticipated. Expressed as a percent change in the mean total number of microspheres over time, we compared the microsphere count with three solutions from after 15 min of perfusion to after 45 min of perfusion (Figure 6). At 4°C using ST solution, more microspheres actually perfused after 45 min with a mean of +136.5%, which significantly differed from the hearts perfused with UW solution by 86% (*p* = 0.037). DCD hearts perfused with UW solution at 4°C experienced a reduction in the number of microspheres perfused by a mean of 50%. At 15°C, there was no difference in the percent reduction of microspheres perfused at 15 min versus 45 min among the three solutions (*p* = 0.273).

### 3.3 Myocardial protection

**FIGURE 7:**
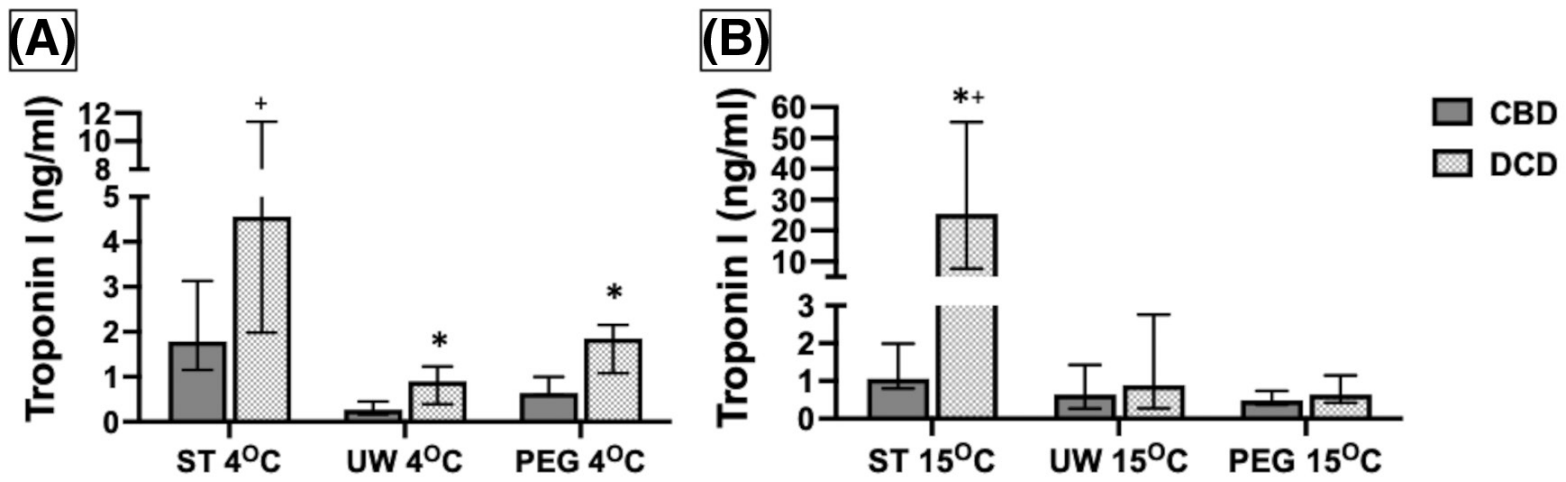
Cardiac troponin I is strikingly elevated for DCD hearts perfused with ST. troponin release for DCD hearts are compared to CBD using the same perfusate (A) at 4°C and (B) at 15°C. CBD, controlled beating‐heart donor; DCD, donation after circulatory death; PEG, polyethylene glycol 20k solution; ST, ST. Thomas solution; UW, University of Wisconsin Belzer solution. Data is expressed as the median troponin concentration in ng/ml. Number of samples per group: CBD ST 4°C *n* = 6, UW 4°C *n* = 6, PEG 4°C *n* = 6, ST 15°C *n* = 6, UW 15°C *n* = 6, PEG 15°C *n* = 6. DCD ST 4°C *n* = 6, UW 4°C *n* = 6, PEG 4°C *n* = 6, ST 15°C *n* = 6, UW 15°C *n* = 6, PEG 15°C *n* = 6. The statistical method used was non‐parametric Wilcoxon rank‐sum test followed by Steel‐Dwass each pair when three groups were compared, and *p* < 0.05 considered significant denoted with an (*) for CBD vs. DCD comparison and (†) for DCD solution comparison. CBD vs. DCD at 4°C using ST (*p* = 0.15), UW (*p* = 0.025), PEG (*p* = 0.01); and at 15°C using ST (*p* = 0.004), UW (*p* = 0.522), PEG (*p* = 0.423). DCD ST vs. UW vs. PEG at 4°C (*p* = 0.005); ST vs. UW (*p* = 0.022), UW vs. PEG (*p* = 0.118), ST vs. PEG (*p* = 0.118). DCD ST vs. UW vs. PEG at 15°C (*p* = 0.005); ST vs. PEG (*p* = 0.014), ST vs. UW (*p* = 0.022), UW vs. PEG (*p* = 0.997). DCD 4°C vs. 15°C ST (*p* = 0.025), UW (*p* = 0.631), and PEG (*p* = 0.025)

The amount of cardiac troponin I (cTnI) released from the hearts was compared to identify a protective benefit of the perfusates from reperfusion injury. The overall median troponin concentrations was compared between CBD and DCD hearts using the same solution at 4 and 15°C, between DCD hearts using the three solutions at 4 and 15°C, and with the same solution comparing 4 and 15°C (Figure 7). A significantly higher level of troponin was released from the DCD hearts compared to CBD hearts at 4°C, especially from hearts perfused with ST solution. At 15°C the troponin release from DCD hearts perfused with UW and PEG solutions was less and comparable to CBD hearts. The highest amounts of troponin release were noted with ST solution both at 4°C and 15°C (*p* = 0.004). When comparing DCD hearts perfused with the three solutions, ST solution at 15°C was associated with highest release of troponin compared to PEG or UW solution (*p* = 0.005). DCD hearts perfused with ST solution had a significantly higher troponin level at 15°C than at 4°C (*p* = 0.025) while PEG had a significantly lower troponin level at 15°C than at 4°C (*p* = 0.025), and UW was comparable at either temperature (*p* = 0.631). These results suggest that DCD hearts sustain significant ischemic damage and when perfused release cTnI. Among the three perfusates we tested in DCD hearts, PEG solution afforded the least troponin release, particularly at 15°C. ST solution appears to afford the least protection for DCD hearts from reperfusion injury.

**FIGURE 8:**
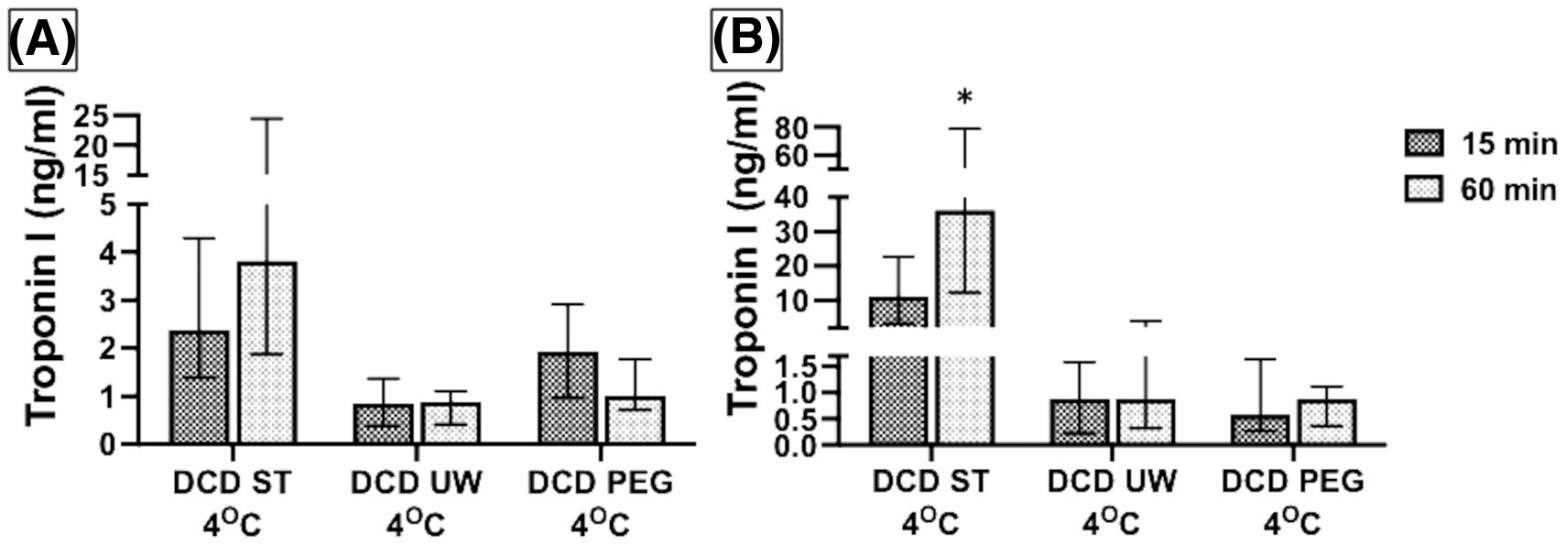
The change in cardiac troponin I level with continued perfusion is significantly elevated for hearts using ST (A) at 4°C and (B) at 15°C, while this affect is not seen in UW or PEG perfused hearts. DCD, donation after circulatory death; PEG, polyethylene glycol 20k solution; ST, ST. Thomas solution; UW, University of Wisconsin Belzer solution. Data is expressed as the median troponin concentration in ng/ml. Number of samples per group: DCD ST 4°C *n* = 6, UW 4°C *n* = 6, PEG 4°C *n* = 6, ST 15°C *n* = 6, UW 15°C *n* = 6, PEG 15°C *n* = 6. The statistical method used was non‐parametric Wilcoxon rank‐sum test followed by Steel‐Dwass each pair when three groups were compared, and *p* < 0.05 considered significant denoted with an (*). DCD ST vs. UW vs. PEG at 4°C (*p* = 0.116). DCD ST vs. UW vs. PEG at 15°C (*p* = 0.005); ST vs. PEG (*p* = 0.014), ST vs. UW (*p* = 0.022), UW vs. PEG (*p* = 0.969)

When the difference in troponin levels from first 15 min of perfusion to the last 15 min of perfusion were compared between DCD hearts with the three solutions (Figure 8), ST solution had the highest release in troponin in the last 15 min, particularly at 15°C compared to UW or PEG solutions (*p* = 0.005). These data suggest that the ST solution is not a suitable preservative solution for DCD hearts and that the reperfusion injury continues to get worse with time.

## 4 DISCUSSION

DCD hearts sustain ischemic damage prior to procurement and cannot be stored in ice for preservation and transportation. Optimal preservation conditions for a DCD heart are not well defined. Our study examined the preservation strategy for the DCD hearts using three non‐blood‐based perfusates at hypothermic and mid‐thermic conditions. We measured perfusion pressure as a surrogate marker of coronary vascular resistance, examined the distribution of perfusate into the myocardial and sub‐endocardial layers with colored microspheres, and measured troponin release in eluate as a marker of ischemia/reperfusion injury. We observed, (1) perfusion pressure was higher for DCD hearts compared to CBD hearts, the difference became statistically significant at 15°C with all three perfusates, (2) among the DCD hearts the lowest rise in perfusion pressure over the duration of perfusion was observed with PEG solution at 15°C, (3) except for PEG solution at 15°C, all other solutions had decreased sub‐endocardial perfusion in DCD hearts, (4) troponin I release from DCD hearts was significantly high with ST solution compared to UW or PEG solution at 15°C, and (5) troponin release in DCD hearts perfused with UW and PEG solutions was comparable to CBD hearts. Overall, the PEG solution at 15°C provided the optimal perfusion and preservation to a DCD heart.

At present, limited number of DCD heart transplantations are done using a machine perfusion system that uses donor whole blood as perfusate.^25^ There are limitations to using whole blood for perfusion; due to multiple organs besides heart are procured, removing a large volume of donor blood takes time and adds to the critical warm ischemia time, potentially jeopardizing the use of other organs. Machine perfusion systems utilize roller pumps, which cause hemolysis and are known to plug capillaries, which may further compromise tissue perfusion.^17^ In addition the blood cells when interacting with the pump tubing promote the release of cytokines that can further damage the donor heart.^26^ Medications administered to the donor, and the high levels of catecholamines which are well known to be present in the donor's blood can have detrimental effects on DCD heart and can lead to primary graft dysfunction. For these reasons, we propose the use of non‐blood‐based preservation solutions for a DCD heart.

We selected the two most commonly used heart preservation solutions, the UW and ST solution, and a novel modified UW solution with PEG‐20K as the impermeant to test DCD heart preservation. Polyethylene glycol (PEG) is a colloid as well as a cell impermeant that affects the oncotic and osmotic gradients at the capillary and cell membrane level, augmenting the expansion of intravascular volume while reducing cellular edema.^26^ In hemorrhagic shock models of low volume resuscitation PEG‐20K based solution has been shown to maintain higher mean arterial pressures, less tachycardia, and better capillary perfusion to the left ventricle, resulting in significantly improved mortality compared to crystalloid based resuscitation.^16^, ^27^, ^28^, ^29^ Based on these qualities we modified UW solution with added PEG‐20K and several substrates for enhanced myocyte metabolism (Table 2) culminating into a novel preservative solution, which we tested, in DCD hearts.

Our previous work showed that the DCD hearts require higher oxygen supply on ex vivo perfusion setup compared to CBD hearts. One of the main limitations of non‐blood‐based perfusate is its inability to deliver enough oxygen to a DCD heart above 15°C.^17^ As DCD hearts do not function when preserved in cold storage (ice) and that a non‐blood‐based perfusates cannot deliver adequate oxygen above 15°C, we designed our study at 15°C that also has an advantage of mid‐thermic preservation.^12^ Cardiomyocyte microtubules subjected to 4°C have increased dysfunction, with elevated basal lactate dehydrogenase release and superoxide production.^30^ Canine DCD hearts stored at 4°C had significantly higher lactate levels and lower left ventricular developed pressure as compared to hearts perfused with warm oxygenated blood.^12^ Cold storage is also associated with cellular swelling from reduced enzymatic activity of ATPases leading to passive intracellular sodium leak.^16^, ^31^ Porcine hearts stored at midthermic (13°C) and sub‐normothermia (21°C) temperatures, showed lower levels of creatinine kinase, cTnI release, and preserved ejection fractions compared to hearts storage at 4°C.^19^ Due to the reasons mentioned above and the inability of non‐blood‐based solutions to support aerobic conditions in DCD hearts above 15°C, we elected to test the three perfusion solutions at 15°C.

Results of this study suggest that perfusion pressures are generally higher for DCD hearts than CBD hearts, regardless of temperature condition or perfusate choice, and that perfusion pressure increases with the duration of perfusion. This is likely attributed to perivascular edema from ischemic injury and vasoconstriction at reduced temperatures. The lowest rise in perfusion pressures over 60 min of perfusion was observed with PEG solution at 15°C, while ST solution had the greatest increase in perfusion pressure over 60 min of perfusion. Although these observations do not reach statistical significance, we favor PEG or UW solution over ST due to the steady rising coronary resistance with the duration of perfusion.

The sub‐endocardial component of myocardium is metabolically more active compared to myocardium. Sub‐endocardial area has terminal capillary flow without watershed boundaries, hence it is more susceptible to ischemia. The capillary density in sub‐endocardium is 10% higher compared to other layers and is maximally dilated at rest; in addition, compressive effects of increased intramyocardial/intraventricular pressure are greater in the sub‐endocardial layer than elsewhere in the myocardium.^32^, ^33^ Adequate perfusion into this layer is, therefore, a strong indicator of optimal perfusion and preservation against reperfusion injury.^13^, ^14^ In our study the DCD hearts perfused with PEG at 15°C had better perfusion into sub‐endocardial layer which was comparable to CBD hearts. UW and ST solutions did not perfuse the sub‐endocardium well. The possible explanations for the better preservation of the DCD hearts perfused with PEG solution are likely the result of superior osmotic and oncotic properties that would allow for efficient capillary perfusion, decreased cellular edema, and less reperfusion injury. In addition, the added amino acids in the PEG solution are easily taken by cardiomyocytes and used in the Krebs cycle to produce electron donors for the electron transport chain in mitochondria. For example, l‐arginine, the amino acid in highest concentration in PEG, specifically has been found to activate nitric oxide synthesis during resuscitation of hemorrhagic shock, which improves arterial flow, decreases lactate production, decreases histologic evidence of reperfusion injury, and improves overall survival in rat studies.^34^

The UW solution uses hydroxyethyl starch as colloid, which imparts higher viscosity at lower temperatures and had poor perfusion through all myocardial layers. ST solution relies on negatively charged chloride ion alone to electrochemically moderate the flow of water but otherwise lacks gluconate or other additives serving as impermeant to prevent cellular swelling and vascular compression. This was evident in our study where the initial low perfusion pressures progressed rapidly to high perfusion pressures and evidence of myocyte breakdown in the form of very high troponin release. Troponin (cTnI) release is an indicator of ischemic myocardial damage. DCD hearts released higher amounts of troponin compared to CBD hearts, especially at 4°C compared to 15°C. DCD hearts perfused with ST solution particularly had the highest concentration of troponin release, both at 4 and at 15°C. Interestingly, DCD hearts perfused with UW and in particular, PEG solution had the least amount of troponin release and was comparable to troponin release from CBD hearts. Our troponin data support DCD heart preservation with PEG solution at 15°C over UW or ST solutions.

A limitation of the study is the adoption of a modified donation after brain death model, in which a fatal traumatic brain injury has not been incurred in our rats. Although our controlled beating‐heart donor (CBD) group mimicked the continued oxygenation and function of cardiac tissue that serves as a premise for transplant suitability, inflicting brain injury can cause a sympathetic storm and systemic inflammatory response that would potentially affect cardiac structure and function.^35^, ^36^ The small sample size in six groups may have limited the statistical power of some reported results. The blood‐based perfusate is physiologic and we did not compare our preservative solution against blood as perfusate. The results from our study can develop future studies investigating cardiac function after midthermic preservation on a Langendorff setup with reanimated hearts or in heterotopic heart transplant murine models.

## 5 CONCLUSION

Our study findings suggest optimal preservation conditions of DCD hearts are with PEG solution at 15°C.

## CONFLICT OF INTEREST

None of the authors has conflicts of interest to disclose.

## AUTHOR CONTRIBUTIONS

Stefano Toldo and Mohammed Quader designed the study. Stefano Toldo and Mohammed Quader secured funding for the study. Renee Cholyway, Stefano Toldo, Oluwatoyin Akande, Kristine Kenning, performed the experiments. Adolfo Gabriele Mauro and Eleonora Mezzaroma developed the imaging and analysis protocol. Renee Cholyway, Stefano Toldo, and Mohammed Quader collected, analyzed, and interpreted the data. Renee Cholyway and Stefano Toldo performed the statistical analysis. Renee Cholyway drafted the article. Stefano Toldo and Mohammed Quader performed a critical revision of the article. All the authors gave final approval of the article.

## Supporting information


Supinfo
