# Supplementary material for: Assessment of machine perfusion conditions for the donation after circulatory death heart preservation
Source: Artif Organs. 2022 Feb 22;46(7):1346–57. doi: 10.1111/aor.14208 (PMC9307000; doi:10.1111/aor.14208)
Supplement: Supplementary file 1 — Supinfo [file AOR-46-1346-s001.docx]

**Supplementary Information:**

**Supplementary Table 1: Distributive Data for Coronary Vascular Resistance**

| **Coronary Vascular Resistance** | | |
| --- | --- | --- |
| **Median Perfusion Pressure** | | |
| **4°C** | **median** | **IQR** |
| CBD ST | 40.9 | (31.54, 53.48) |
| DCD ST | 71.1 | (36.75, 105) |
| CBD UW | 50.0 | (36.66, 61.47) |
| DCD UW | 66.7 | (58.75, 73.59) |
| CBD PEG | 63.1 | (53.44, 79.31) |
| DCD PEG | 82.4 | (38.63, 87.97) |
| **15°C** | **median** | **IQR** |
| CBD ST | 27.6 | (8.34, 39.72) |
| DCD ST | 57.4 | (49.75, 81.31) |
| CBD UW | 63.3 | (42.69, 69.51) |
| DCD UW | 81.3 | (74.16, 102.88) |
| CBD PEG | 34.8 | (23.16, 46,31) |
| DCD PEG | 62.7 | (47.28, 80.25) |
| **For Change in Mean Perfusion Pressure** | | |
| **4°C** | **mean** | **SD** |
| DCD ST 15min | 41.25 | 17.72 |
| DCD ST 60min | 101 | 64.54 |
| DCD UW 15min | 53.25 | 8.42 |
| DCD UW 60min | 77.33 | 12.04 |
| DCD PEG 15min | 52.2 | 21.85 |
| DCD PEG 60min | 80.75 | 42.42 |
| **15°C** | **mean** | **SD** |
| DCD ST 15min | 37.67 | 9.43 |
| DCD ST 60min | 90.5 | 34.89 |
| DCD UW 15min | 66.83 | 14.6 |
| DCD UW 60min | 106.67 | 32.98 |
| DCD PEG 15min | 56.42 | 16.27 |
| DCD PEG 60min | 71 | 28.14 |

**Supplementary Table 2: Distributive Data for Distribution of Perfusate**

| **Distribution of Perfusate** | | |
| --- | --- | --- |
| **Median E/M Ratio** | | |
| **4°C** | **median** | **IQR** |
| CBD ST | 0.19 | (0.11, 0.23) |
| DCD ST | 0.02 | (0.01, 0.09) |
| CBD UW | 0.15 | (0.12, 0.23) |
| DCD UW | 0.00 | (0, 0.002) |
| CBD PEG | 0.17 | (0.06, 0.23) |
| DCD PEG | 0.00 | (0.002, 0.07) |
| **15°C** | **median** | **IQR** |
| CBD ST | 0.14 | (0.04, 0.22) |
| DCD ST | 0.02 | (0.003, 0.07) |
| CBD UW | 0.25 | (0.16, 0.30) |
| DCD UW | 0.04 | (0.004, 0.09) |
| CBD PEG | 0.13 | (0.05, 0.23) |
| DCD PEG | 0.04 | (0.008, 0.14) |
| **For Change in Mean Total Beads** | | |
| **4°C** | **mean** | **SD** |
| DCD ST 15min | 59.39 | 35.9 |
| DCD ST 45min | 75.7 | 55.23 |
| DCD UW 15min | 23.60 | 14 |
| DCD UW 45min | 10.51 | 4.24 |
| DCD PEG 15min | 71.40 | 62.04 |
| DCD PEG 45min | 39.44 | 18.57 |
| **15°C** | **mean** | **SD** |
| DCD ST 15min | 56.67 | 18.16 |
| DCD ST 45min | 56.50 | 26.81 |
| DCD UW 15min | 26.21 | 13.77 |
| DCD UW 45min | 23.63 | 19.97 |
| DCD PEG 15min | 40.38 | 17.3 |
| DCD PEG 45min | 18.19 | 16.45 |

**Supplementary Table 3: Distributive Data for Myocardial Protection**

| **Troponin Release** | | |
| --- | --- | --- |
| **Median Troponin Level** | | |
| **4°C** | **median** | **IQR** |
| CBD ST | 1.79 | (1.15, 3.13) |
| DCD ST | 4.56 | (1.98, 11.38) |
| CBD UW | 0.27 | (0.16, 0.45) |
| DCD UW | 0.90 | (0.39, 1.23) |
| CBD PEG | 0.64 | (0.59, 1.00) |
| DCD PEG | 1.85 | (1.08, 2.15) |
| **15°C** | **median** | **IQR** |
| CBD ST | 1.06 | (0.80, 1.99) |
| DCD ST | 25.42 | (7.63, 55.18) |
| CBD UW | 0.64 | (0.27, 1.43) |
| DCD UW | 0.89 | (0.27, 2.76) |
| CBD PEG | 0.49 | (0.37, 0.74) |
| DCD PEG | 0.64 | (0.42, 1.15) |
| **For Change in Median Troponin** | | |
| **4°C** | **median** | **IQR** |
| DCD ST 15min | 2.38 | (1.39, 4.29) |
| DCD ST 60min | 3.82 | (1.88, 24.43) |
| DCD UW 15min | 0.86 | (0.38, 1.37) |
| DCD UW 60min | 0.89 | (0.41, 1.11) |
| DCD PEG 15min | 1.93 | (0.97, 2.91) |
| DCD PEG 60min | 1.02 | (0.72, 1.77) |
| **15°C** | **median** | **IQR** |
| DCD ST 15min | 11.22 | (3.23, 22.27) |
| DCD ST 60min | 36.20 | (12.22, 78.83) |
| DCD UW 15min | 0.89 | (0.22, 1.59) |
| DCD UW 60min | 0.88 | (0.32, 3.92) |
| DCD PEG 15min | 0.58 | (0.27, 1.64) |
| DCD PEG 60min | 0.88 | (0.36, 1.12) |

*E/M=sub-endocaridal to myocardial space ratio, CBD=controlled beating-heart donor, DCD=donor after circulatory death, ST=St. Thomas, UW=University of Wisconsin Machine Perfusion Solution, PEG=Polyethylene glycol-20k Machine Perfusion Solution
